# Monocrotophos pesticide affects synthesis and conversion of sex steroids through multiple targets in male goldfish (*Carassius auratus*)

**DOI:** 10.1038/s41598-017-01935-6

**Published:** 2017-05-23

**Authors:** Hua Tian, Yang Sun, Hui Wang, Xin Bing, Wei Wang, Shaoguo Ru

**Affiliations:** Marine Life Science College, Ocean University of China, Qingdao, 266003 China

## Abstract

Monocrotophos (MCP) is an organophosphorus pesticide that is median-toxic to fish. MCP pesticide resulted in an increase of 17 beta estradiol following a decrease in testosterone in male goldfish (*Carassius auratus*). To fully understand the mechanism of MCP pesticide that causes the imbalance between male and female hormones, we determined the levels of plasma cholesterol, spermatic steroidogenic acute regulatory protein mRNA, steroidogenesis enzyme mRNA, plasma sex hormone synthesis intermediates, and effectual hormones in male goldfish exposed to MCP pesticide at nominal concentrations of 0.01, 0.10, and 1.00 mg/L for 21 days in a semi-static exposure system. The results indicated that MCP pesticide (a) led to decreased steroidogenic acute regulatory protein mRNA levels; (b) decreased mRNA levels of cholesterol side chain cleavage enzyme and cytochrome P450 17 alpha hydroxylase, which are steroidogenesis enzymes involved in androgen synthesis; and (c) increased cytochrome P450 aromatase mRNA levels, a steroidogenesis enzyme involved in the synthesis of effectual estrogen. The present study provides evidence that MCP pesticide affects synthesis and conversion of sex steroids through multiple targets in male goldfish.

## Introduction

Monocrotophos (MCP, CAS number 6923-22-4) is an organophosphorus pesticide that is high-toxic to birds, median-toxic to fish, and listed as a UNEP Prior Informed Consent chemical. Production, management, and use of MCP pesticides have been comprehensively banned in China since January 1, 2007, but it is still extensively detected in China and some other developing countries. MCP was detected in snow pea samples from western China in 2010^1^. MCP residues in brinjal, okra, cucurbits, crucifers, and green chilies were 0.023–1.140 mg/kg in the Andaman Islands, India^2^, and its concentration in the industrial wastewater near Lucknow City, India, was 8.32 ± 3.9 μg/L^3^.

In our previous studies, it was demonstrated that MCP pesticide was a potential environmental estrogen^4^, and it was furthermore determined that MCP pesticide induced mRNA expression of aromatizing enzyme in the gonads of male goldfish (*Carassius auratus*) and resulted in an increase of 17 beta estradiol (E_2_) following a decrease in testosterone (T)^5^. The changes in the E_2_ and T levels were in good agreement with the increase in aromatase expression; however, this might not be the only mechanism by which MCP pesticide disrupts the balance of sex hormones in male fish because a series of enzymatic reactions are involved in sex hormone synthesis in teleosts.

In theory, the process of sex hormone synthesis provides numerous potential targets for MCP pesticide. First, cholesterol serves as precursor to all steroid hormones, and some xenobiotics disturbed the synthesis of sex hormones by affecting cholesterol levels^6–8^. Second, the synthesis of sex hormones starts only when cholesterol is transported to the inner mitochondrial membrane. Steroidogenic acute regulatory protein (StAR) plays an important role in this delivery process, and thus, low StAR levels will lead to a sharp decline or even interruption of sex hormone synthesis. For example, beta sitosterol exposure changed gonadal transcript levels of StAR in goldfish, leading to lower concentrations of T^9^. Third, after being transported to the inner mitochondrial membrane, cholesterol is translated into pregnenolone under the catalysis of the cholesterol side chain cleavage enzyme (CYP11A1/P450scc). The conversion from pregnenolone to effectual hormones requires the participation of 3 beta hydroxysteroid dehydrogenase (3 beta HSD), cytochrome P450 17 alpha hydroxylase (CYP17/P450c17/P45017alpha), 17 beta hydroxysteroid dehydrogenase (17 beta HSD), cytochrome P450 aromatase (CYP19/P450arom), 20 beta hydroxysteroid dehydrogenase (20 beta HSD), cytochrome P450_11beta_ (CYP11beta/P450_11beta_), and 11 beta hydroxysteroid dehydrogenase (11 beta HSD), all of which are potential target sites of xenobiotics. Govoroun *et al*. demonstrated that E_2_ inhibited spermatic P450c17, 3 beta HSD, and P450_11beta_ gene expression in rainbow trout (*Oncorhynchus mykiss*)^10^. MCP pesticide might affect synthesis and conversion of sex hormones *via* a number of pathways, such as changing the contents of synthesis substrates or influencing gene expression and activities of steroidogenesis enzymes.

This study was conducted to fully understand the mechanism by which MCP pesticide causes an imbalance between male and female hormones in male goldfish. First, effects on plasma total cholesterol (TC), high-density lipoprotein cholesterol (HDL-C), and low-density lipoprotein cholesterol (LDL-C) levels were examined to determine whether a lack of substrates for sex hormone synthesis is responsible for inhibited T levels. Second, spermatic StAR mRNA levels were quantified, which could indicate the influence of MCP on the transport of cholesterol from the outer to the inner mitochondrial membrane. Third, mRNA levels of eight kinds of steroidogenesis enzymes and plasma levels of six kinds of sex hormone synthesis intermediates and four kinds of effectual hormones were determined, to elucidate the effects of MCP pesticide on steroidogenesis in male goldfish.

## Results

### Effects of MCP pesticide on gonadosomatic index (GSI)

**Figure 1:**
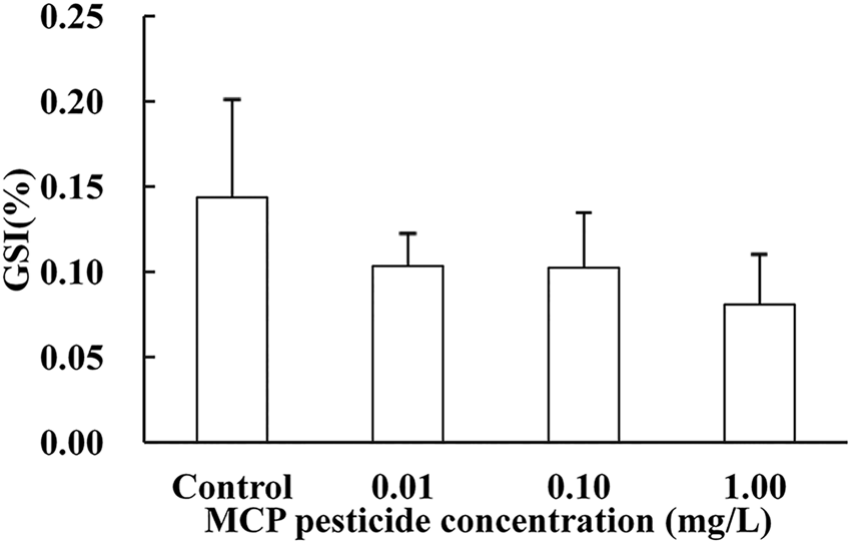
Effects of MCP pesticide on GSI of male goldfish (*Carassius auratus*).

The fishes were sampled in summer with degraded gonads. GSI of the control male goldfish was 0.14 ± 0.06%, which was not different from that in any of the MCP pesticide treatments studied (Fig. 1).

### Effects of MCP pesticide on cholesterol levels

**Figure 2:**
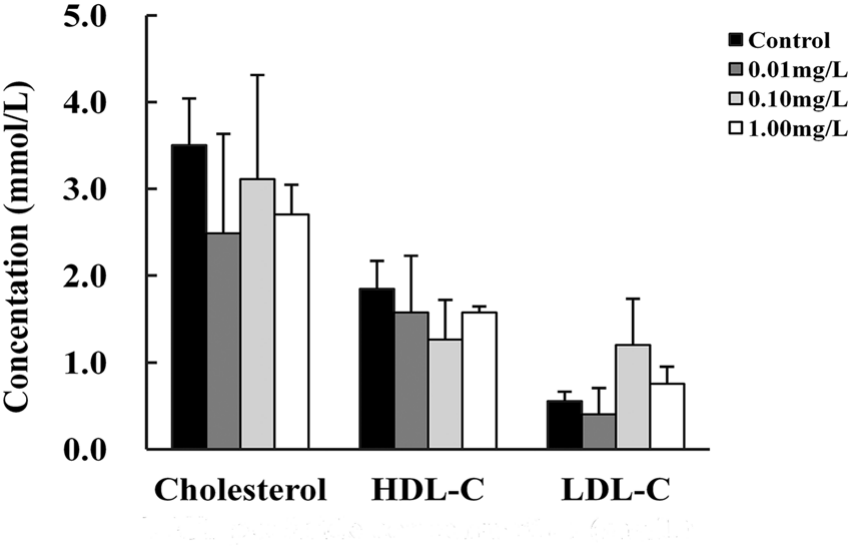
Effects of MCP pesticide on total cholesterol, HDL-C, and LDL-C content in plasma of male goldfish (*Carassius auratus*).

In the control group, the content of plasma HDL-C was higher than that of LDL-C. TC, HDL-C, and LDL-C levels were not influenced by MCP pesticide exposure (Fig. 2).

### Effects of MCP pesticide on gene expression of StAR and steroidogenesis enzymes

Fragments encoding P450scc, 3-beta-HSD, 20-beta-HSD, 17-beta-HSD1, P450_11beta_, and 11-beta-HSD2 genes of goldfish were amplified and cloned. The deduced amino acid sequences showed high homology to those of other fish species, containing typical P450 conserved features as indicated in Table 1 ^11–14^.

**Table 1:** Alignment of the deduced amino acid sequences of goldfish steroidogenesis enzyme genes with those of other animal species.

Gene	GenBank No.	Amino acid sequence homology	Conserved domain
P450scc	JQ340311	91% (*Gobiocypris rarus*)	
		84% (*Tautogolabrus adspersus*)	
		81% (*Oryzias latipes*)	
		75% (*Danio rerio*)	
3-beta-HSD	JQ867353	91% (*Gobiocypris rarus*)	Partial sequence of cofactor binding motif: Gly–X–X–Gly–X–X–Gly^11^; Enzyme active site of short-chain alcohol dehydrogenase: Tyr–X–X–X–Lys
		87% (*Danio rerio*)	
		77% (*Oncorhynchus mykiss*)	
		76% (*Clarias gariepinus*)	
20-beta-HSD	KC193327	97% (*Cyprinus carpio*)	Adenine ring binding domain: X-Asp-X-X-Asp; Sequence for a *β* sheet: Gly-Gly-X-Asp-X-X-X-Asn-Asn-Ala-Gly-X; Active site: Thr-Asn-X-X-Gly-Thr-X-X-X-X-X; Substrate binding site: X-X-Asn-X-Ser-Ser-X; Enzyme active site: Tyr-X-X-X-Lys^12, 13^; Sequence regulating hydrogen bonds in nicotinamide ring: X-X-X-Asn-X-X-X-Pro-Gly-X-X-X-Thr^14^
		90% (*Danio reri*o)	
		80% (*Tachysurus fulvidraco*)	
		79% (*Oncorhynchus mykiss*)	
17-beta-HSD1	JX036034	86% (*Danio rerio*)	Coenzyme binding domain: Thr-Gly-X-X-X-Gly-X-Gly; Connection between coenzyme binding domain and substrate activation site: Asn-Ala-Gly; Enzyme active site: Tyr–X–X–Ser–Lys with Ser and Asn upstream; Sequence III directing reaction
		71% (*Anguilla japonica*)	
		70% (*Oreochromis niloticus*)	
P45011beta	KC193328	85% (*Danio rerio*)	Steroid binding region; Oxygen binding region; Ozols’ region
		81% (*Oreochromis niloticus*)	
		80% (*Oryzias latipes*)	
		74% (*Oncorhynchus mykiss*)	
11-beta-HSD2	KC193326	91% (*Danio rerio*) 82% (*Anguilla japonica*) 81% (*Oncorhynchus mykiss*) 80% (*Clarias gariepinus*) 78% (*Oryzias latipes*)	Coenzyme I binding domain: Gly-Phe-Gly; Conserved sequence of 11-beta-HSDs: Cys-Met-Glu-Val-Asn-Phe-Phe-Gly; Enzyme active site: Tyr-Gly-X-Ser-Lys-Ala-Ala

**Figure 3:**
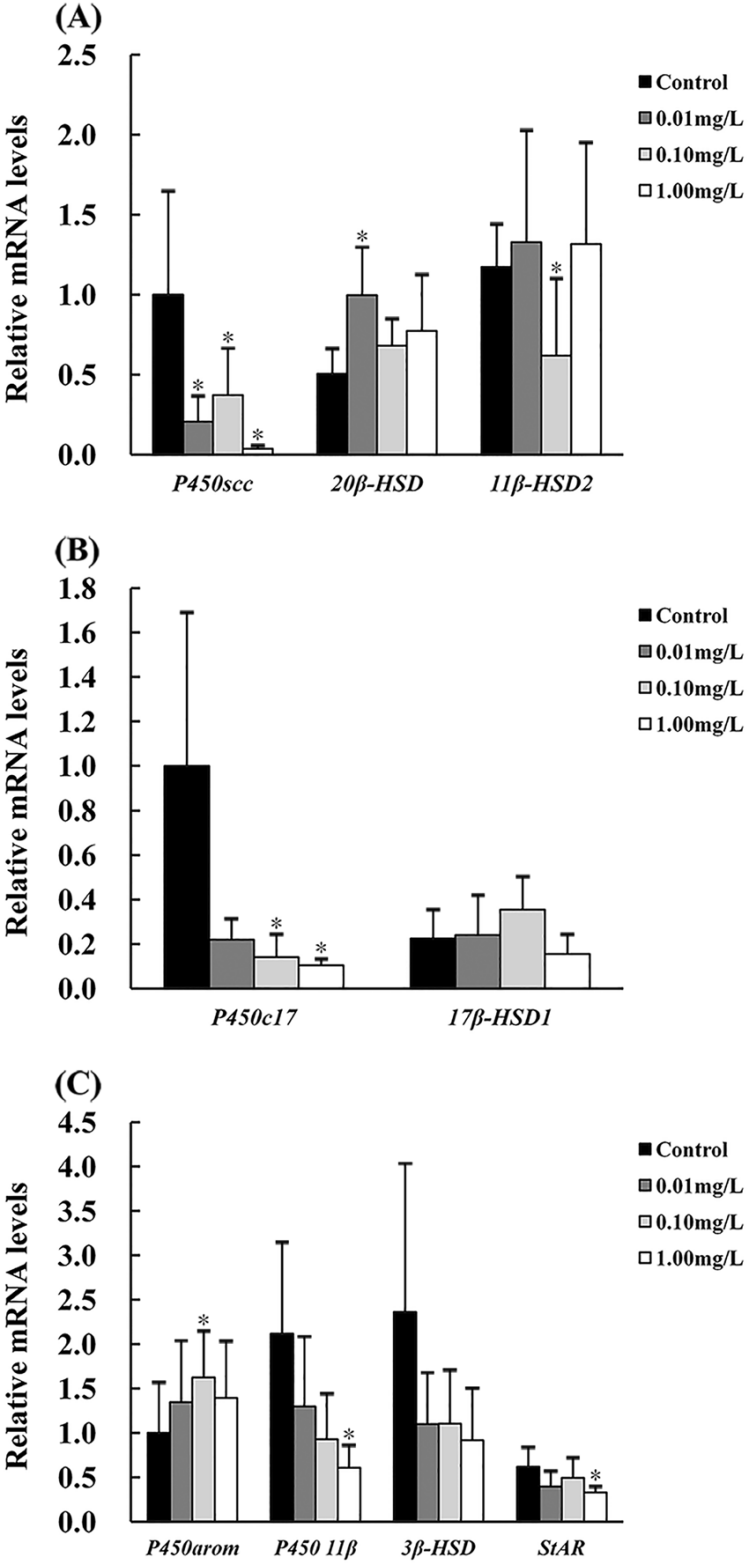
Effects of MCP pesticide on testicular transcriptional levels of StAR and steroidogenesis enzymes in male goldfish (*Carassius auratus*).

Gene expression levels of eight steroidogenesis enzymes, as well as StAR were detected. A significant decrease in StAR gene transcriptions was caused by MCP pesticide exposure (*P* < 0.01, η^2^ > 0.15, Fig. 3C). Transcription levels of P450scc, which is related to synthesis of pregnenolone, were significantly down-regulated in all three exposure groups (*P* < 0.01, η^2^ > 0.15, Fig. 3A). Significant down-regulation of P450c17, which is a key enzyme involved in androstenedione synthesis, was also observed (*P* < 0.01, η^2^ > 0.15, Fig. 3B), whereas 3-beta-HSD mRNA levels exhibited no significant changes (Fig. 3C). Gene expression of steroidogenesis enzymes involved in synthesis of effectual androgen and estrogen was decreased and increased, respectively, by MCP pesticide exposure. Specifically, expression of P450_11beta_ (*P* < 0.01, η^2^ > 0.15, Fig. 3C) and 11-beta-HSD2 (*P* < 0.01, η^2^ > 0.15, Fig. 3A) was down-regulated after MCP pesticide treatment, and P450arom A mRNA levels were significantly up-regulated in the 0.10 mg/L exposure group (η^2^ > 0.15, Fig. 3C), although 17-beta-HSD1 mRNA levels were not significantly changed in any exposure group. The conversion of 17 alpha hydroxyprogesterone to 17 alpha, 20 beta dihydroxy-4-pregnen-3-one is catalyzed by 20-beta-HSD, and only the 0.01 mg/L exposure group showed up-regulation of its expression (*P* < 0.01, Fig. 3A).

### Effects of MCP pesticide on plasma sex hormone levels

**Figure 4:**
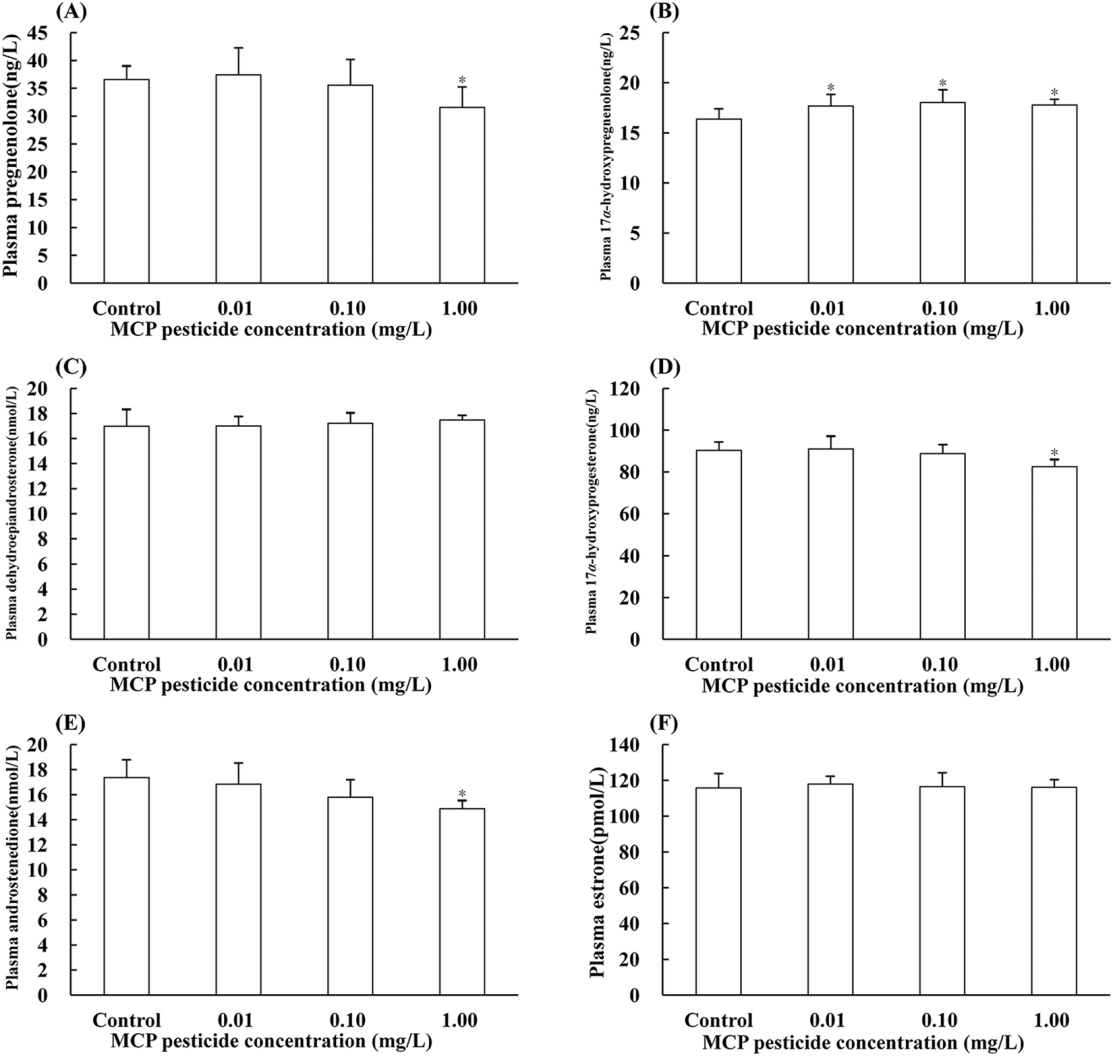
Effects of MCP pesticide on plasma content of intermediate hormones in male goldfish (*Carassius auratus*).

**Figure 5:**
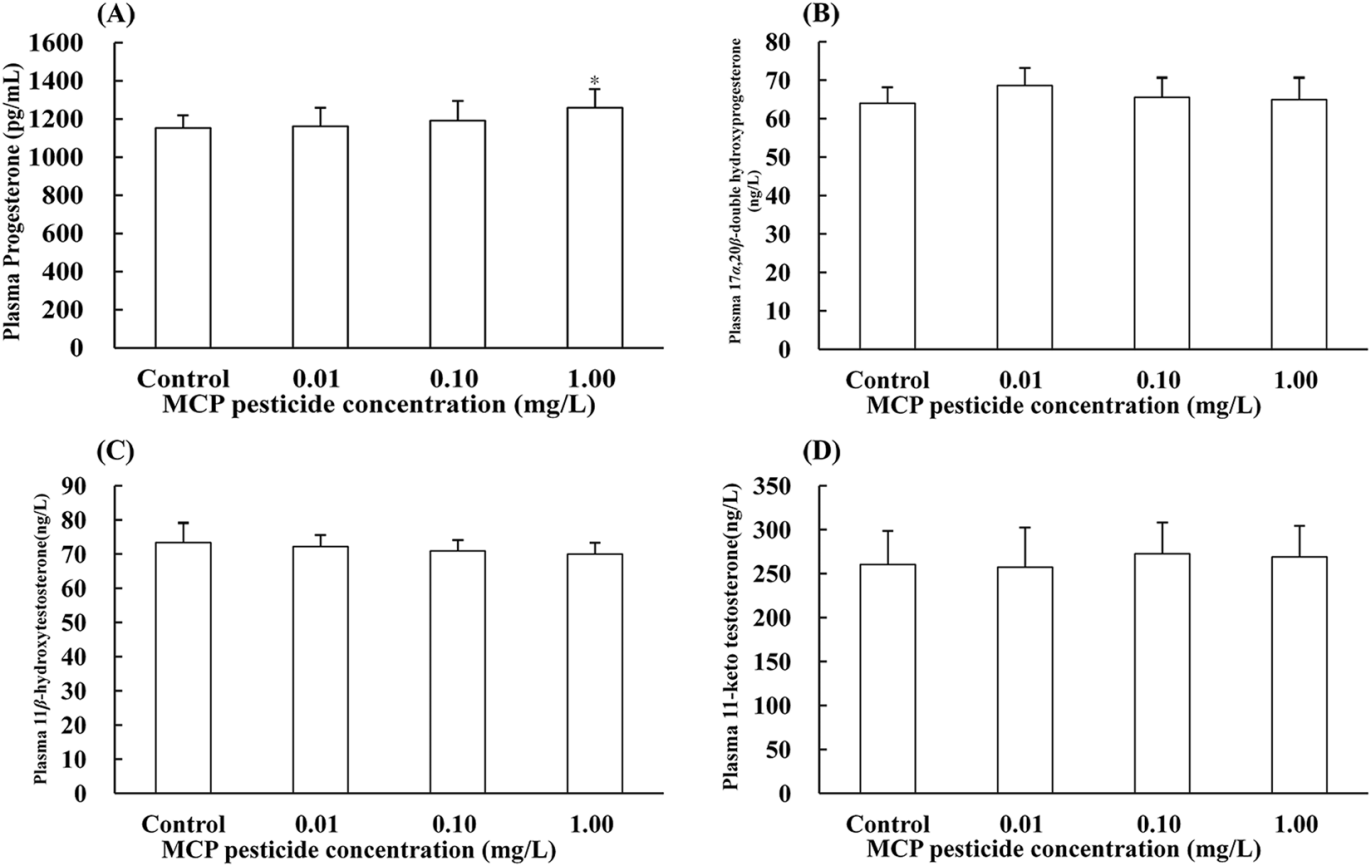
Effects of MCP pesticide on plasma content of effectual hormones in male goldfish (*Carassius auratus*).

Effects of MCP pesticide on plasma levels of six kinds of intermediates, as well as four kinds of effectual hormones were determined. The highest pesticide concentration (1.00 mg/L) significantly decreased plasma pregnenolone levels (*F*_3,36_ = 4.413, *P* < 0.05, η^2^ > 0.15, Fig. 4A). Androstenedione is an important precursor of male and female sex hormones, and decreases in the levels of plasma androstenedione (*P* < 0.01, η^2^ > 0.15, Fig. 4E) and one of its substrates, 17 alpha hydroxyprogesterone (*P* < 0.01, η^2^ > 0.15, Fig. 4D), were detected; however, dehydroepiandrosterone, which is another substrate for androstenedione, exhibited no significant changes in any of the three exposure groups (Fig. 4C). Plasma 17 alpha hydroxypregnenolone (*F*_3,36_ = 5.826, *P* < 0.05, η^2^ > 0.15, Fig. 4B) and progesterone (*F*_3,36_ = 3.020, *P* < 0.05, η^2^ > 0.15, Fig. 5A) levels, which are also involved in synthesis and transformation of androstenedione, were increased by pesticide exposure, with significant changes detected only in the highest dose group for progesterone. There were no significant changes in 17 alpha, 20 beta double hydroxyprogesterone (Fig. 5B), 11 beta hydroxytestosterone (Fig. 5C), 11-keto testosterone (11-KT) (Fig. 5D), or estrone (Fig. 4F) levels in any of the groups tested.

## Discussion

**Figure 6:**
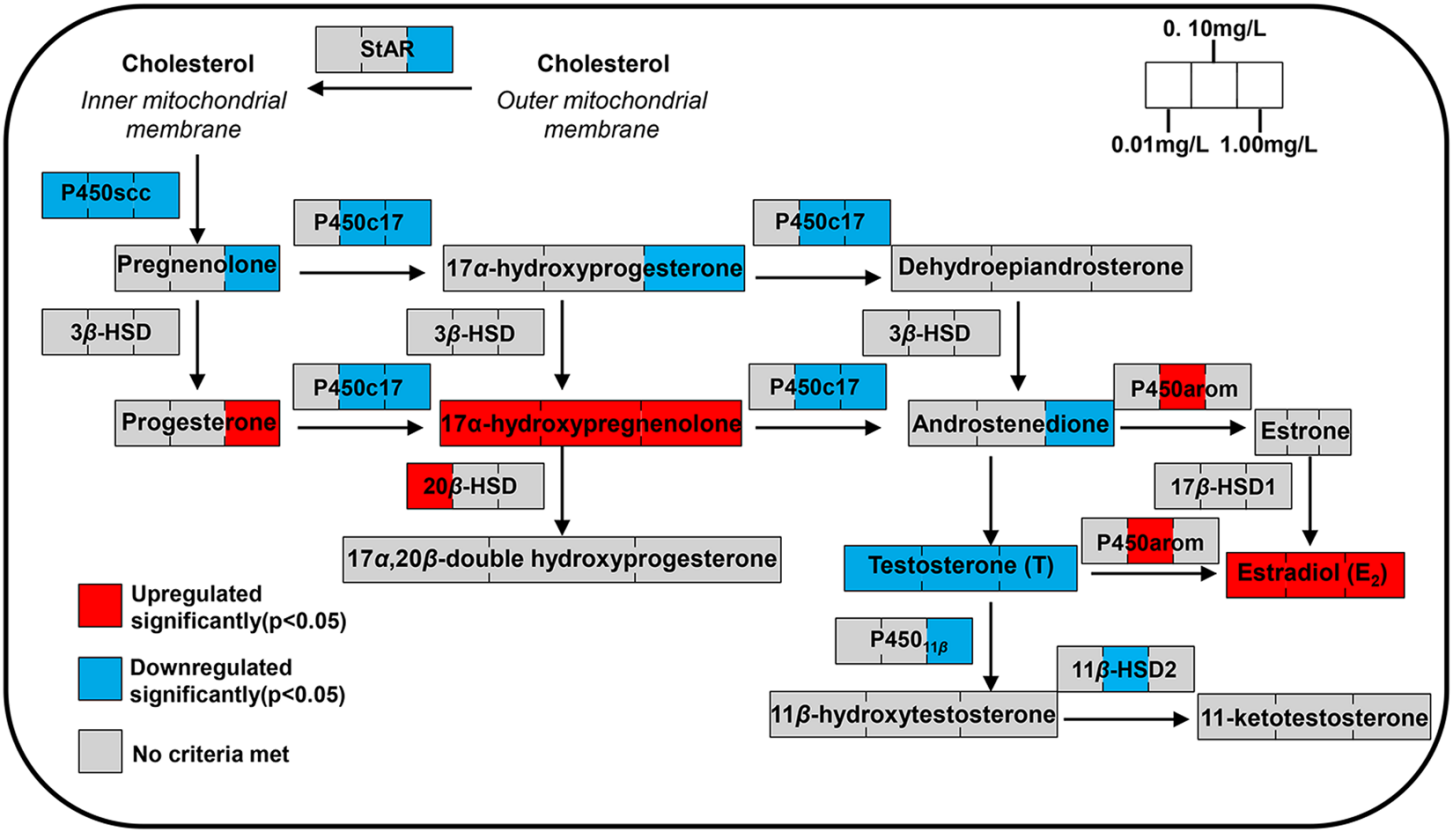
Outline of effects of MCP pesticide on steroidogenesis in male goldfish (*Carassius auratus*).

In this study, mechanisms by which MCP pesticide affects sex hormone synthesis and conversion of male goldfish were discussed from four aspects: (a) cholesterol levels, (b) gene expression of StAR and steroidogenesis enzymes; (c) intermediate products of the process of sex hormone synthesis; and (d) levels of effectual hormones (Fig. 6).

In bony fish, the substrates of steroid synthesis are mainly provided by exogenous cholesterol^11^. For example, climbing perch (*Anabas testudineus*) used plasma cholesterol as the raw material of sex hormone synthesis^15^. Low-density lipoproteins (LDL) play a leading role in mammals, but most cholesterol was transported by high-density lipoproteins (HDL) in fish^7, 16, 17^. In this study, plasma levels of LDL-C and HDL-C were both maintained following MCP pesticide exposure, suggesting that the content of raw material for sex hormone synthesis was not affected.

StAR can transfer cholesterol to the inner mitochondrial membrane, providing a reaction substrate for synthesis of pregnenolone, which is catalyzed by P450scc. In rainbow trout, StAR transcriptional levels were closely related to changes in plasma steroid hormones^18^. P450scc catalyzes the first step of steroidogenesis, making it a key enzyme during this process. MCP pesticide inhibited gene expression levels of StAR in male goldfish testes, leading to the reduction of cholesterol that was transported to the inner mitochondrial membrane, and the decrease in plasma levels of pregnenolone caused by MCP pesticide exposure is consistent with the inhibition of P450scc gene expression.

Pregnenolone, 17*α*-hydroxypregnenolone, and dehydroepiandrosterone can be converted to progesterone, 17*α*-hydroxyprogesterone, and androstenedione, respectively. These reactions were all catalyzed by 3-beta-HSD. P450c17 is one of the key enzymes of the steroidogenesis pathway in teleostean gonads. There are two kinds of P450c17 in bony fish: P450c17-I and P450c17-II. P450c17-I shows both 17*α*-hydroxylase and 17,20-lyase activity, whereas P450c17-II has only 17*α*-hydroxylase activity. Pregnenolone and progesterone can be converted to 17*α*-hydroxypregnenolone and 17*α*-hydroxyprogesterone, respectively, by 17*α*-hydroxylase activity; 17,20-lyase activity can break the chemical bonds between C17 and C20 and transform 17*α*-hydroxypregnenolone into dehydroepiandrosterone or 17*α*-hydroxyprogesterone into androstenedione. We found a significant reduction of plasma T in male goldfish after MCP pesticide exposure in our previous study^5^, and we observed that MCP pesticide exposure resulted in decreased plasma androstenedione in this study, which is the substrate of T. The down-regulation of androgens by MCP pesticide corresponded to decreases in P450c17 mRNA levels.

Estradiol is formed stepwise, starting with aromatization of androstenedione to estrone, followed by conversion of estrone to E_2_ *via* 17-beta-HSD activity, or conversion of androstenedione to T *via* 17-beta-HSD, followed by aromatization of T to E_2_. The intermediates androstenedione and estrone have low bioactivity, whereas T and E_2_ are effectual hormones with high bioactivity. MCP pesticide increased P450arom A gene expression in male goldfish gonads, and this is consistent with our previous study^5^.

P450_11beta_ catalyzes T into 11-beta-hydroxytestosterone^19^, and 11-beta-HSD2 further converts 11-beta-hydroxytestosterone into 11-KT^20^. The reduction of P450_11beta_ gene expression led to inhibition of 11-KT synthesis^10, 21^, whereas Kusakabe *et al*. suggested that plasma 11-KT levels had less relevance to 11-beta-HSD2 gene expression in the testes^22^. In this study, we observed significant declines of 11-beta-HSD2 and P45011beta mRNA levels, but there was no significant change in 11-KT.

Androgen is responsible for development of the testes. Based on the appearance and histologic features of the testes, the development process in goldfish can be classified into six periods^23^. Individuals were sampled in August in this study, when testes had degraded to period III after spermiation, and GSI of the control male goldfish was as low as 0.14 ± 0.06%. Although an abnormally low plasma concentration of T was observed concomitant with an increase in MCP pesticide concentration in our previous study^5^, GSI was not affected in this study. This may be explained by the unchanged 11-KT levels, because in general 11-KT, rather than T, is the dominant androgen in most fish species, including goldfish.

Studies on endocrine disruption of exogenous compounds, particularly regarding steroidogenesis, have focused mainly on gene expression and activity of steroidogenesis enzymes^24, 25^, especially aromatase^26, 27^, although multiple factors could be involved in steroidogenesis. In this research, we demonstrated that MCP pesticide acted on many target sites, affecting the transport of cholesterol and gene expression of steroidogenesis enzymes, leading to abnormal levels of sex hormone synthesis intermediates and effectual hormones, and thus resulting in a proportional imbalance of sex hormones in male goldfish.

## Materials and Methods

### Fish exposure and sample protocol

Goldfish (8.4 ± 0.6 cm standard length; 17.6 ± 3.6 g wet mass) were obtained from a local dealer in Qingdao, China. Following acclimation in the laboratory at 24–26 °C and under a 16 h light/8 h dark cycle for two weeks, fish were exposed to 0.01, 0.10, and 1.00 mg/L MCP pesticide (40% water-soluble preparation), the concentrations of which were 1/10000, 1/1000, and 1/100 of the 96-h LC_50_ (about 100 mg/L, our unpublished results), respectively^5^. Experiments were conducted in 70 L aquaria containing 50 L dechlorinated tap water using a semi-static toxicity test. Twenty liters of water was renewed daily to keep the MCP concentrations constant. Fish were fed a non-estrogenic pelletized diet daily. Additionally, the fish were handled according to the National Institute of Health Guidelines for the handling and care of experimental animals. The animal utilization protocol was approved by the Institutional Animal Care and Use Committee of the Ocean University of China.

After 21 days of exposure^28–33^, goldfish were anesthetized in 75 mg/L MS-222 (Sigma-Aldrich, St. Louis, MO, USA). Blood was taken from the caudal vein using chilled heparinized syringes. After centrifugation (700 *g*, 15 min, 4 °C or 2810 *g*, 10 min, 4 °C), plasma was frozen at −80 °C for cholesterol or hormone quantification. Individuals were identified as male or female during dissection. The testes were weighed and related to body weight to determine GSI (GSI = gonad weight/body weight × 100%), and then gonad samples were frozen in liquid nitrogen and stored at −80 °C for steroidogenesis enzyme gene quantification. In addition, ovaries and testes collected from unexposed fish were frozen in liquid nitrogen and stored at −80 °C for steroidogenesis enzyme gene cloning.

### Cholesterol quantification

After twice dilution with 0.7% saline, plasma was analyzed using a fully automatic biochemistry analyzer to determine the content of TC, HDL-C, and LDL-C, with commercial kits obtained from Shanghai Fenghui Medical Science and Technology Co., LTD, Shanghai Rongsheng Biological Pharmaceutical Co., LTD, Beijing Beihua Kangtai Clinical Reagent Co., LTD, and Shenzhen-Mindray Biological Medical Electronics Co., LTD, China, respectively. The assay detection ranges were 0–20 mmol/L for TC, 0.05–6.0 mmol/L for HDL-C, and 0–9 mmol/L for LDL-C, respectively. The inter- and intra-assay coefficients of variation for TC and LDL-C were ≤5%. The inter- and intra-assay coefficients of variation for HDL-C were ≤2.5% and ≤4.0%, respectively.

### cDNA fragment cloning and mRNA quantification of steroidogenesis enzyme genes

Isolation of total RNA from gonad tissue was accomplished using phenolic reagent TRIzol (Invitrogen, Carlsbad, CA, USA) according to the manufacturer’s protocol. Extracted RNA was measured by spectrometry at OD_260/280_ prior to being treated with DNase I (Promega, Madison, WI, USA). M-MLV first strand reverse transcription reaction I (Invitrogen, Carlsbad, CA, USA) was built with 1.0 μL Oligo(dT)_12–18_ (500 μg/mL), 5.5 μL total RNA, 1.0 μL 10 mM each dNTP, and 4.5 μL diethypyrocarbonate (DEPC) water, incubated at 65 °C for 5 min, and then rapidly frozen. Reverse transcription reaction II was built with 4.0 μL 5× first strand synthesis buffer, 2.0 μL 0.1 mol/L DTT, 1.0 μL cloned ribonuclease inhibitor (40 U/μL, Takara Bio Inc., Shiga, Japan), and 12.0 μL reverse transcription reaction I, hatched at 37 °C for 2 min, and then hatched at 37 °C for 50 min after 1 μL M-MLV reverse transcriptase (200 U/μL) was added. The reaction was terminated by heating the reaction mixture at 70 °C for 15 min.

To clone cDNA fragments of steroidogenesis enzyme genes, including P450scc, 3-beta-HSD, 20-beta-HSD, 17-beta-HSD1, P450_11beta_, and 11-beta-HSD2, from goldfish gonads, primers were designed for conserved amino acid sequence regions based on alignments of related gene sequences in other fish species that are closely related to goldfish (Table 2). Primers designed by Primer Premier 5.0 were compounded by Sangon Biological Engineering Technology & Services Co., Ltd., Shanghai, China. The polymerase chain reaction (PCR) was conducted with an initial denaturation step at 94 °C for 5 min, followed by 40 cycles at 94 °C for 30s, annealing temperature (Table 2) for 30s, 72 °C for 1 min, and a final extension step at 72 °C for 5 min. Amplified PCR products were cloned in pGM-T easy vector (Takara Bio Inc., Shiga, Japan) and sequenced (Sangon Biological Engineering Technology & Services Co., Ltd., Shanghai, China). MegAlign and GeneDoc programs in DNAStar were used to analyze the homology of the deduced amino acid sequences of goldfish steroidogenesis enzymes with those of other vertebrates.

**Table 2:** Primers for PCR amplification.

Gene	Primer	Primer sequence (5′~3′)	Annealing temperature (°C)	Amplicon size (bp)
P450scc	Sense	GTNYTGTATGGGGARCGTYTG	50.6	≈500
	Anti-sense	CWGGATGTAAYCTBAGWGTTTC		
3-beta-HSD	Sense	TGTGTGTGGTGACAGGAGCC	60.0	824
	Anti-sense	CCCAAAGCCTAATGGTGAAAG		
20-beta-HSD	Sense	TGGCGATTGTGAAAGGGTTG	56.6	690
	Anti-sense	CTTCCTCTGGACTCTTGGTAGCAT		
17-beta-HSD1	Sense	GGATTGGACTCAGCCTTGC	58.4	698
	Anti-sense	CATCGCCTCCAGATACACCT		
P450_11beta_	Sense	GAGCTGTCACGTTCTGTACGGC	63.5	532
	Anti-sense	TGTCTCCTTGATGGTCCCCTTC		
11-beta-HSD2	Sense	TTCAGGTTTTGGTAATGCTACA	53.0	697
	Anti-sense	GTCGATGACAGGACTCAGGTC		

N = A/T/C/G, W = A/T.

Quantitative real-time PCR assays were developed to examine the expression patterns of steroidogenesis enzyme genes and StAR in response to MCP pesticide based on the cloned goldfish steroidogenesis enzyme cDNA fragments and StAR, P450c17, P450arom A, beta actin (internal control 1), and 18S rRNA (internal control 2) sequences for goldfish published in GenBank. Primers designed by Primer Premier 5.0 were compounded by Sangon Biological Engineering Technology & Services Co., Ltd., Shanghai, China (Table 3). Real-time PCR was performed in 20 μL reaction mixtures consisting of SYBR^®^ Premix Ex *Taq* ^TM^ II (Takara Bio Inc., Dalian, China), 0.4 μL ROX Reference Dye II (Takara Bio Inc., Shiga, Japan), 0.8 μL sense primer (10 μM), 0.8 μL anti-sense primer (10 μM), 4.0 μL cDNA, and 4.0 μL ultrapure water (sterile). The real-time PCR reactions were incubated at 95 °C for 30s, followed by 40 cycles of 95 °C for 5s, and 60 °C for 30s. Melting curve analyses were conducted to differentiate between the desired PCR products and primer-dimers or DNA contaminants. In addition, 2% agarose gel electrophoresis of the PCR products was performed to confirm the presence of single amplicons of the correct predicted size (data not shown). Gene expression was measured in duplicate. Neither beta actin nor 18S rRNA levels were affected by any of the experimental conditions in the study. The relative target gene mRNA levels were normalized using the geometric mean of the beta actin and 18S rRNA gene expression levels following the formula 2^−ΔΔCt^ and plotted on a logarithmic scale^34^.

**Table 3:** Primers for real-time PCR amplification.

Gene	GenBank No.	Primer	Primer sequence (5′~3′)	Amplicon size (bp)
StAR	AY877430	Sense	CGAGAGTGCCAATGGTGATAAGGTG	93
		Anti-sense	TCTGTCTTCTGTTCCAGCATCACTTCC	
P450c17	FJ809936	Sense	CCGACCTGTCGCCCCACT	193
		Anti-sense	GCAGCACAAACCATCACCCTC	
P450arom A	AB009336	Sense	TGCTGGGTCTGGGTCCTCTT	102
		Anti-sense	CCCGCACAATGTCTCCGTAT	
P450scc	JQ340311	Sense	GAGGCTTGGGACGGCATCTTTA	107
		Anti-sense	GCCAGCACACCAGGGTACTTCT	
3-beta-HSD	JQ867353	Sense	TGCGAAGAGCGTGTAAAGGAGC	193
		Anti-sense	GGATTGGGACCAGCCACCTC	
17-beta-HSD1	JX036034	Sense	TGCTGTCCATCTCGCATCAAAT	163
		Anti-sense	GTCAAGTATAGACTGCTGGTCGGTC	
20-beta-HSD	KC193327	Sense	TGCGGTTGCTGGACTGAAATCTG	173
		Anti-sense	GCTCTGTTGCTGAATGTTTGAAGGC	
P45011beta	KC193328	Sense	ATCCTGGGTGTGCTGGGTCAG	145
		Anti-sense	GGTTCCTCGCTAACTCGAAGAGAG	
11-beta-HSD2	KC193326	Sense	AGGTAGACATCACCCAGCCTCAGC	114
		Anti-sense	TGTTCACACACCATCCAGCGTTATT	
beta-actin	AB039726	Sense	GAAACTGGAAAGGGAGGTAGC	115
		Anti-sense	CTGTGAGGGCAGAGTGGTAGA	
18S rRNA	FJ710820	Sense	CGAGACGAGCCACCACCTATC	148
		Anti-sense	CGGTATTCAGCGGCGACAG	

### Hormone quantification

Fish hormone ELISA Kits (R&D Systems, Inc., Minneapolis, MN, USA) were used to quantify plasma pregnenolone, progesterone, 17 alpha hydroxypregnenolone, 17 alpha hydroxyprogesterone, 17 alpha, 20 beta double hydroxyprogesterone, dehydroepiandrosterone, androstenedione, estrone, 11 beta hydroxytestosterone, and 11-KT levels according to the manufacturer’s instructions. The concentration of each hormone was calculated from the linear part of the standard curve, and plasma was diluted when the hormone concentration exceeded the linear range.

### Statistics

All data were tested for normality and homoscedasticity. Kruskal-Wallis H test followed by Dunnett’s correction was used to analyze GSI, TC, HDL-C, LDL-C, dehydroepiandrosterone, 17 alpha hydroxyprogesterone, androstenedione, estrone, 3-beta-HSD, 11-beta-HSD2, 20-beta-HSD, P450scc, P450c17, P45011beta, and StAR data for an abnormal distribution or heteroscedasticity. A one-way analysis of variance (ANOVA) followed by Tukey’s test was adopted for statistical analyses of effectual hormones and the other two intermediates, as well as steroidogenesis enzymes 17-beta-HSD1 and P450arom, because all assumption were met for parametric analyses. Effect size was also considered during statistical analysis. η^2^ was calculated as η^2^ = SS_effect_/SS_total_ for parametric analysis and η^2^ = H/(N−1) for Kruskal-Wallis tests. By convention, 0.01, 0.06, and 0.15 are considered “small”, “medium”, and “large”-effect sizes, respectively.
